# Micro-Volume Blood Separation Membrane for In-Situ Biosensing

**DOI:** 10.3390/bios12090712

**Published:** 2022-09-02

**Authors:** Qin Zhu, Huimin Wu, Zhen Ma, Yuqiao Liu, Junmin Li, Ling Zhu, Xinran Zhang, Chengcheng Wang, Dajing Chen, Danhua Zhu

**Affiliations:** 1 State Key Laboratory for Diagnosis and Treatment of Infectious Diseases, The First Affiliated Hospital, Zhejiang University School of Medicine, Hangzhou 311100, China; 2 School of Pharmacy, Hangzhou Normal University, Hangzhou 311121, China; 3 MOE Key Laboratory of Macromolecular Synthesis and Functionalization, Department of Polymer Science and Engineering, Zhejiang University, Hangzhou 310027, China

**Keywords:** blood separation, porous membranes, colorimetric assay

## Abstract

In this paper, we report a point-of-care (POCT) testing strip based on a porous membrane structure for whole blood separation and colorimetric analysis without external supporting equipment. Conventional blood tests rely on large instruments for blood pretreatment and separation to improve measurement accuracy. Cellulose acetate (CA) membranes with different pore diameters and structures were prepared via a non-solvent method for the separation of whole blood. Among them, CA@PEG-2000 membranes with nano-pores on the surface and micro-pores in the interior facilitated the capture of blood cells on the surface, as well as the free diffusion of plasma through the porous interior structure. The fluid flow of blood in the asymmetric porous structure can be theoretically estimated using the Lucas-Washburn equation. Compared with the conventional paper strips and other porous membranes, the CA@PEG-2000 membrane with an immobilized sensing layer exhibited efficient blood separation, a short response time (less than 2 min), an ultralow dosage volume (5 μL), and high sensitivity. The fabricated blood separation membranes can be further used for the detection of various biomarkers in whole blood, providing additional options for rapid quantitative POCT tests.

## 1. Introduction

Blood is one of the most crucial biological samples for medical diagnosis, containing plasma and cells circulating in the body [1]. In practice, plasma usually needs to be separated from whole blood to ensure sensitivity and selectivity in measurements, as cells in whole blood are complex and unstable components [2,3,4,5]. In addition, the natural redness of the hemoglobin in whole blood interferes with the optical and fluorescent signal reading [6,7,8,9]. It is therefore necessary to rapidly separate blood cells from whole blood for accurate diagnoses [10,11]. The traditional procedure employed by laboratories to extract plasma is centrifugation, which relies on large equipment and significant amounts of blood samples [12,13]. Point-of-care tests (POCT) need to deliver results in a short period of time without relying on large laboratory instruments, while achieving sensitivity and portability at the same time is a key technical challenge in clinical diagnostics. Microfluidic technology is an emerging method to separate nano- to microliter volumes of blood cells by unique channel designs, including flexographic printing, lithography, inkjet printing, screen printing and chemical modification [14,15]. However, these microfluidic separation strategies require complex fabrication procedures and specialized instrumentation, increasing the inconvenience and costs of POCT devices.

The conventional separation method is a time-consuming process that requires blood volumes in microliters. Recently, many researchers have reported using porous structures to filter out red blood cells (6–8 µm) and leukocytes (12–17 µm) from whole blood samples. Dai et al. reported a nanometer-scale three-dimensional porous-structure hydrogel, allowing the diffusion of small analytes such as glucose, while blocking much larger blood cells without sample pretreatment [16]. Park et al. reported a three-dimensional printing method for fabricating a plasma separation membrane based on paper microfluidic analytical devices (3D-μPADs) without additional assembly after printing [17]. Wu et al. presented the fiber-based microfluidic concept of “lab on hollow fiber membrane” for sample size sieving and rapid biological assays quantification [18,19]. In lateral flow systems, it is still of great significance to study the effects of porous structures on blood separation performance.

**Scheme 1:**
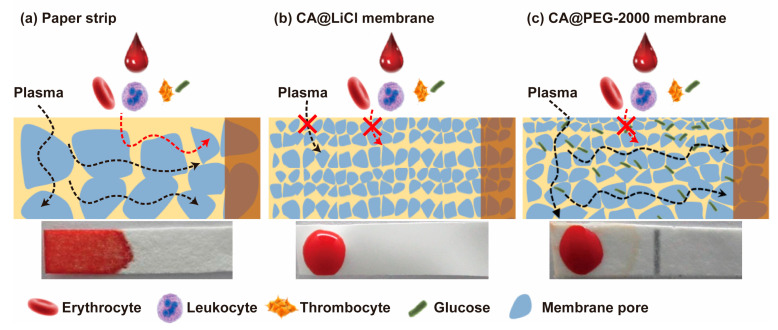
The schematic diagrams of different membranes and bioassay strips.

In this paper, we verified the influence of phase separation temperature and solvents (Table S1), and developed a lateral flow testing strip based on porous CA membranes for whole blood separation and analysis. As depicted in Scheme 1, we fabricated various CA membranes with different porous structures, including a unique asymmetric porous structure (Scheme 1C) that was fabricated for in-situ whole blood separation and colorimetric biosensing. In the asymmetric CA membranes, the nano-pores on the surface and micro-pores in the interior facilitated the retention of blood cells on the surface and the free diffusion of plasma through the interior porous structure. The total separation and reaction time of the CA@PEG-2000 membrane is less than 2.5 min, and the glucose detection range covers the concentration range of clinical blood samples. Compared with traditional paper strips and other porous membranes, this asymmetric porous membrane exhibited excellent blood plasma separation and color rendering performance. The prepared blood separation membranes can be expanded to detect many other biomarkers in whole blood, providing more options for rapid quantitative POCT assays.

## 2. Materials and Methods

### 2.1. Materials and Instruments

Glucose oxidase (GOx, 100 U·mL^−^^1^) from Aspergillus niger was supplied by Sangon Biotech Co., Ltd. (Shanghai, China). Cellulose acetate (CA), lithium chloride (LiCl), polyethylene glycol (PEG-2000), methanol, starch, and tetrahydrofuran (THF) were purchased from Aladdin Reagent Co., Ltd. (Shanghai, China). Ethyl cellulose, polyethylene glycol, sodium chloride and proclin were purchesd from Sinopharm Chemical Reagent (Shanghai, China). All solutions were prepared with Milli-Q purified water. Whole blood samples were obtained from the Affiliated Hospital of Hangzhou Normal University and collected in clinic, and stored in the EDTA blood collection tube in a refrigerator at 4 °C until used. This work collected thirty clinic samples in the whole test, but twenty of them were used to verify the blood separation performance, while ten were used in glucose concentration tests. All experiments involving blood samples were approved by the Human Ethics Committee of Hangzhou Normal University (Ethics approval number: HZ2021-1109).

A field emission-scanning electron microscope (FE-SEM, Hitachi-S4800, Tokyo, Japan) was used to observe the structure and morphology of cellulose acetate membranes. The membranes were freeze-fractured in liquid nitrogen and we observed the pore distribution in the cross-section view. Fourier transform infrared (FT-IR) spectra were taken on a Thermo Scientific Nicolet iN10 (Waltham, MA, USA). Microscope images were obtained using an inverted optical microscope (Nikon, TS2, Tokyo, Japan) in the bright field mode. The UV-vis absorption spectra of the chromogenic reaction were acquired by a UV-Vis spectrometer (UV2550, Shimadzu, Kyoto, Japan).

### 2.2. Fabrication of Cellulose Acetate Membranes

Cellulose acetate membranes with different pore structures were prepared via non-solvent-induced phase separation. (1) CA membranes with a no-pore structure on the surface: 7 wt. % CA was added to 200 mL THF. (2) CA@LiCl membranes with a foam structure: 7 wt. % CA and 6 wt. % LiCl were added to 200 mL THF. (3) CA@PEG-2000 membranes with an asymmetric structure: 7 wt. % CA and 6 wt. % PEG-2000 were added to 200 mL THF. All solutions were magnetically stirred at room temperature for 48 h, coated on the clean glass plate and then immersed in water for 30 min at 40 °C. Lastly, all fabricated membranes were dried at 50 °C to remove the remaining organic solvent.

### 2.3. Fabrication of Cellulose Acetate Membranes for Blood Glucose Detection

The whole assembly process of the strip: 0.5 g of starch was dissolved in 100 mL milli-Q-water and then heated for complete dissolution. Next, 0.1 M KI and 1% GOx were added into the starch solution. Finally, the mixture was deposited in a 2 mm wide line on the membranes by a dual-axis dispensing system (Dexin TL771). The deposited area was dried at room temperature and acted as the colorimetric reagent for glucose detection in the separated blood.

### 2.4. Colorimetric Detection of the Unseparated Blood Cells

An occult blood kit was used to evaluate the blood separation performance. Here, 5 μL of whole blood was dropped on one end of the porous membranes. The membranes with plasma diffusion parts were cut off and immersed in the occult blood kit solution. The unseparated red blood cells in the diffusion part will react with the occult blood kit and generate a colorimetric change, which could be measured by a UV-Vis spectrometer at 567 nm. All tests were performed five times.

### 2.5. Colorimetric Detection of the Blood Glucose

Approximately 5 μL of blood sample was dropped on one end of the membrane. The dense surface of the cellulose acetate membrane can block blood cells, while the micro-porous sublayer allows the diffusion of plasma containing small analytes such as glucose by micro-capillary action. For each glucose concentration level, three testing strips were measured and averaged. All blood samples were calibrated with a YSI biochemical analyzer. The intensity levels of blood glucose can be visually recognized by the naked eye and recorded with a camera. Colorimetric assays were implemented in a light control box and we analyzed the intensity with Image J software. For each measurement, we used the measure function in Image J and selected a 25 × 120 pixel image area for intensity measurement.

The glucose simulation solution with viscosity similar to blood was prepared by adding ethyl cellulose (3 wt. %), polyethylene glycol (3.5 wt. %), sodium chloride (0.85 wt. %) and proclin (0.03 wt. %) in DI water. The linear calibration plot (Figure S1) of the chromogenic intensity vs. the glucose concentration using Image J software (Image J 1.52a, Wayne Rasband, National Institutes of Health, Bethesda, MD, USA) was established by detecting standard glucose concentrations.

## 3. Results and Discussion

### 3.1. Characterization of CA Membranes

**Figure 1:**
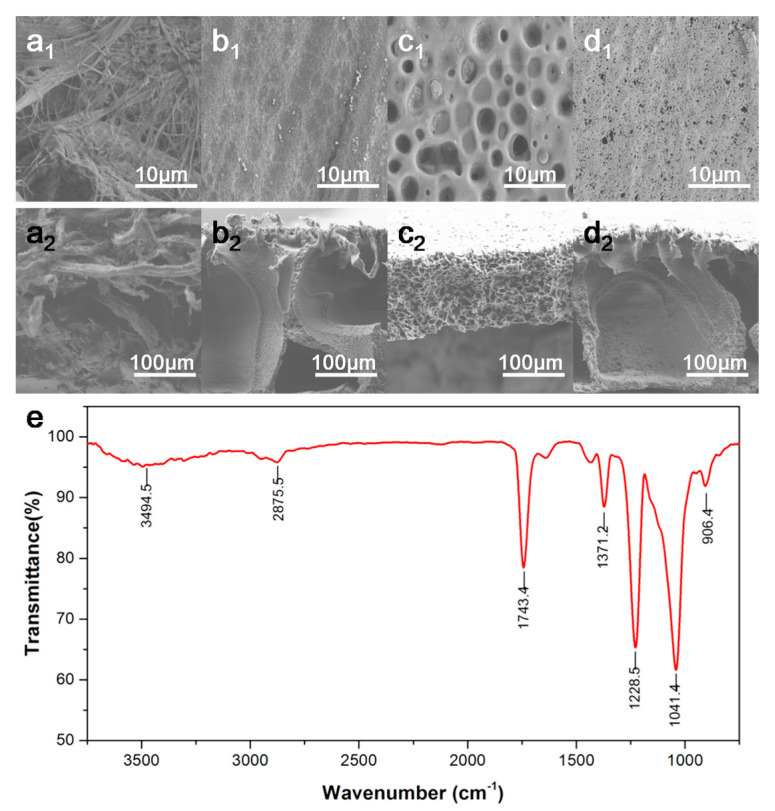
SEM images of the surface (**a_1_**,**b_1_**,**c_1_**,**d_1_**) and cross-section (**a_2_**,**b_2_**,**c_2_**,**d_2_**) of (**a**) a paper strip purchased from Whatman (England) with a bore diameter of 11 μm, (**b**) CA membranes, (**c**) CA@LiCl membranes, and (**d**) CA@PEG-2000 membranes. (**e**) FTIR spectra of CA@PEG-2000 membranes.

The morphology and pore size of different membranes are shown in Figure 1. As shown in the surface and cross-section SEM images (Figure 1a_1_,a_2_), the paper strip exhibited a disordered pore structure with variable pore diameters incapable of filtering out the red blood cells. Among the various CA membranes, different pore structures and sizes were observed. As indicated in Figure 1b_1_,b_2_, there were no pores on the surface of the pure CA membrane, which prevented blood from entering the interior of the porous membrane. CA@LiCl membranes and CA@PEG-2000 membranes showed distinct porous structures compared with the pure CA membranes. Adding additives, such as LiCl, increased the coagulation rate and formed membranes with high interconnectivity and porosity [20]. PEG acting as a pore former was proposed to increase the hydrophilic property of the membrane [21,22,23]. The CA@LiCl membranes showed a foam structure with a small pore diameter averaging 2 µm (Figure 1c_1_,c_2_). In contrast, CA@PEG-2000 membranes had an asymmetric structure, while small pores on the surface captured blood cells and large pores in the sublayer facilitated the liquid capillary flow (Figure 1d_1_,d_2_). The pore diameter in the interior part of the CA@LiCl membranes was significantly lower than that of CA@PEG-2000 membranes, indicating poor liquid hydrodynamic performance.

The FTIR spectra of CA@PEG-2000 membranes are shown in Figure 1e. The broad band around 3650-3450 cm^−^^1^ represents the stretching of O−H, and the band due to C−H was located around 3000–2850 cm^−^^1^. The strong peak at 1743.4 cm^−^^1^ in the FTIR spectrum could be attributed to the formation of new hydrogen bonds between the O−H groups of CA and the C=O bond of PEG-2000; the presence of these interactions implies excellent miscibility of CA and PEG-2000 in the blend membranes [24,25]. The presence of PEG-2000 in CA@PEG-2000 membranes resulted in bands at 1100 cm^−^^1^ characteristic of PEG-2000 ether groups. The peak at 1371.2 cm^−^^1^ indicates the bending of C−H, and 1228.5 cm^−^^1^ is for the acetate group of the C−O bond, and C−O−C groups at 1041.4 cm^−^^1^ characteristic of CA were observed [26].

### 3.2. Theoretical Analysis of Blood Plasma Separation Membranes by Liquid Diffusion

The pore diameter of the conventional paper strip was over 10 μm and did not match the scale of red blood cell separation, making it incapable of detecting analytes in the whole blood samples without pretreatment. Therefore, whole blood separation membranes should have appropriate pore distributions that can sieve blood cells (7–8 μm), while promoting liquid diffusion by self-driven capillary force.

In order to achieve highly efficient blood separation performance, the fabricated membranes must have appropriate pore sizes on their surface to filter blood cells, as well as larger pores (a less tortuous path) in the sublayer to facilitate plasma capillary flows.

The fluid flows in a porous medium can be theoretically estimated using the Lucas-Washburn equation [27]. This equation assumes cylindrical pores, unlimited dosage volume, and negligible gravitational effects. The theoretical equation of fluid flow is as follows:

(1) $$L\left(t\right)=\sqrt{\frac{\Upsilon dcos\theta }{4\mu }}\frac{\sqrt{t}}{\tau }$$

Fluid with (liquid-vapor) surface tension (*γ*) and viscosity (*μ*) imbibes a distance *L* in time (*t*), while *d* is defined as the capillary diameter, and *θ* is the contact angle between the fluid and the capillary wall. The parameter tortuosity (*τ*) describes the tortuousness of streams/tubes in the porous network and is usually defined as *τ* = *L_t_*/*L*_0_, where L_t_ is the length of a tortuous line, and L_0_ is the length of a straight line in a tortuous path.

The theoretical results demonstrate that the lower tortuosity (larger pores) facilitate the liquid flow in the membranes. In order to achieve good blood separation performance, the fabricated membranes need to have appropriate pore sizes on their surface to capture blood cells, as well as larger pores (less tortuous path) in the sublayer to facilitate plasma flows along the membranes.

### 3.3. Blood Separation of the CA@PEG-2000 Membranes

**Figure 2:**
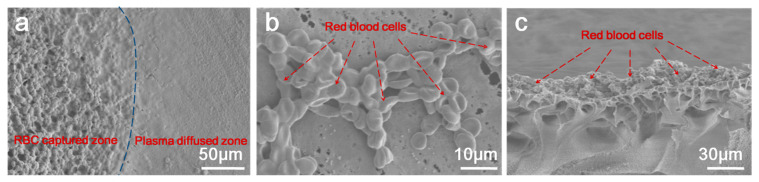
SEM images of the (**a**,**b**) surface and (**c**) cross-section of CA@PEG-2000 membranes after blood cell separation. The dotted blue line represents the dividing line between blood cells and plasma.

**Figure 3:**
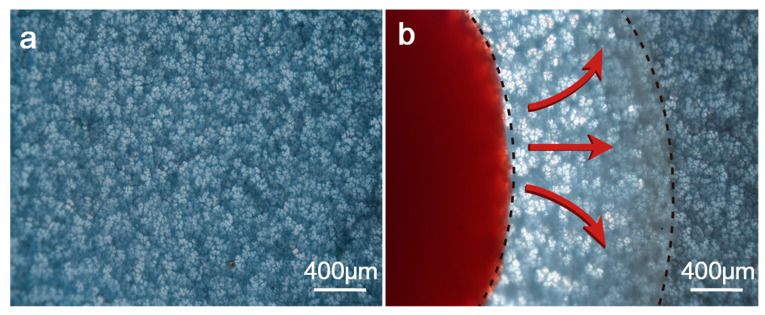
Zoom-stereo microscope images of (**a**) CA@PEG-2000 membranes and (**b**) 5 µL of whole blood separated by CA@PEG-2000 membranes.

The morphologies of the surface and cross-section of CA@PEG-2000 membranes after blood separation were studied by SEM, and are in Figure 2. The blood cells were trapped on the surfaces of CA@PEG-2000 membranes due to the size sieving effect of micropores on the membrane surface. In the magnified SEM images (Figure 2b), the red blood cells retained their original morphology and did not rupture at separation. As shown in Figure 2c, the red blood cells were trapped on the upper surface and did not enter the interior of the porous membrane, indicating that the micropores on the surface acted as the separation layer. Furthermore, the interior part had larger pores that allowed for the diffusion of analytes such as glucose via micro-capillary and liquid diffusion. According to the microscope images in Figure 3, the plasma diffused through the porous membrane by capillary force, and the liquid filled the pores, causing a change in light transmission.

**Figure 4:**
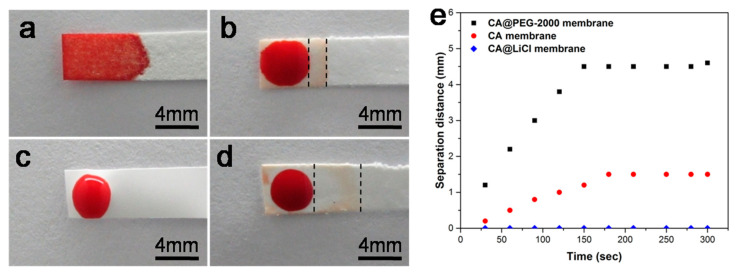
The diffusion distances of whole blood samples in (**a**) a paper strip, (**b**) CA membranes, (**c**) CA@LiCl membranes, and (**d**) CA@PEG-2000 membranes, (**e**) The diffusion distances-time relationship of whole blood samples in CA membranes, CA@LiCl membranes, and CA@PEG-2000 membranes.

The diffusion distances and times of whole blood samples in different CA membranes are the most critical factors in improving colorimetric detection accuracy and speed. The whole blood diffusion distances in the paper strip, CA membranes, CA@LiCl membranes, and CA@PEG-2000 membranes are illustrated in Figure 4. The paper strips with 10–20 μm pore diameters cannot filter out the red blood cells. The diffusion distances of CA membranes and CA@PEG-2000 membranes are 1.5 mm and 4.5 mm, respectively, indicating their ability for both size separation and capillary permeation. The viscous whole blood could not infiltrate into the membranes with small pores by gravity, resulting in a diffusion distance of 0 mm for CA@LiCl membranes. Furthermore, the diffusion time of the whole blood samples in the pure CA membranes and CA@PEG-2000 membranes is 2.5 min (Figure 4e), indicating a rapid blood separation performance. In summary, the CA@PEG-2000 membranes demonstrated highly efficient and rapid blood separation performances, which could be applied in clinical blood sample analyses. We performed a comparison of our testing strip with other passive separation devices in recent reports, as shown in Table S2. From this table, we can see that the overall performance of our testing strip is on par with state-of-the-art technologies.

### 3.4. Bioassay Application in Occult Blood and Human Blood Samples

**Figure 5:**
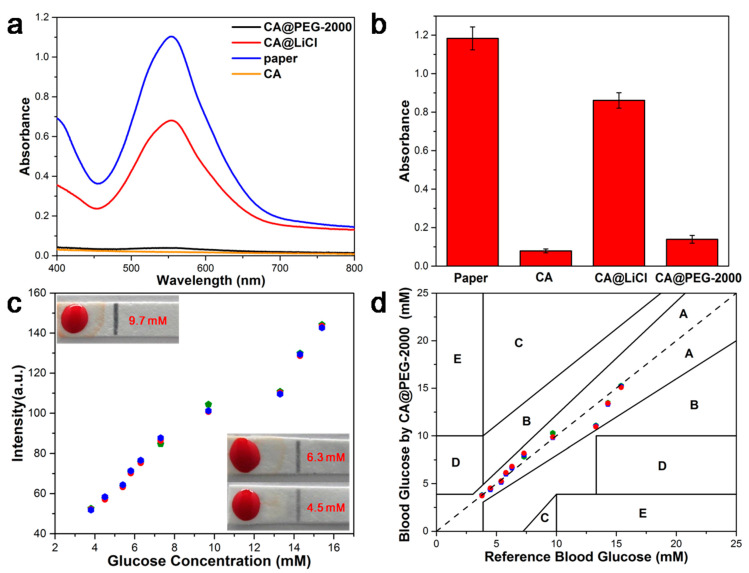
(**a**) UV-Vis absorption spectra of the chromogenic reaction with the different membranes. (**b**) Test repeatability of different membranes at 567 nm (standard deviation, *n* = 5). (**c**) The chromogenic intensity (figures with physical pictures). (**d**) Error grid analysis of real human blood samples in CA@PEG-2000 membranes.

To verify the blood separation performance, we measured the residual hemoglobin in the plasma using an occult blood kit. The membrane containing separated plasma was cut and immersed in the occult blood kit solution. The hemoglobin in the unseparated blood cells will catalyze the oxidation of TMB in the presence of hydrogen peroxide, causing the kit solution to turn blue. The higher the absorption peak at 567 nm, the more residual hemoglobin remained on the membrane, implying poor blood separation performance. In Figure 5a, both paper and CA membranes showed high absorption peaks, while CA@PEG-2000 membranes exhibited very low absorbance under 0.1 (close to the absorbance of free Hb in plasma), indicating its higher blood cell separation efficiency.

The glucose measurements in clinical blood samples are shown in Figure 5c. Approximately 5 μL of blood was dropped on CA@PEG-2000 membranes and diffused to the colorimetric detecting zones of GOx, KI and starch. GOx catalyzed the oxidation of blood glucose to H_2_O_2_ and gluconic acid. H_2_O_2_ oxidized KI to yield I_2_, and the starch reacted with I_2_ to produce a purple color. In the practical application, the blood cells were filtered out on the surface, while smaller molecules diffused freely through the pores by liquid diffusion. The response times of analytes and colorimetric assay reagents were within 30 s. CA@PEG-2000 membranes were used to measure the blood samples with different glucose concentrations, and the reaction was followed by quantifying the chromogenic intensity using Image J software. As shown in Figure 5c, the intensity of the colorimetric reaction area increased with the increasing glucose concentration in samples. The glucose concentrations in the samples were all calibrated using a YSI biochemical analyzer. Compared with the results of the CA@PEG-2000 membranes and the YSI standard method, 30 data points are located in Zone A of Figure 5d, indicating the measured values deviated from the standard values within 20%. Zone A (no risk) represents glucose values that deviate from the reference by no more than 20%, while upper and lower zone B (no or benign treatment) represents values that deviate from the reference by >20%, zone C (likely to affect clinical) shows values that would result in overcorrecting acceptable blood glucose levels, zone D (could have significant medical risk) represents “dangerous failure to detect and treat” errors, and Zone E (could have dangerous consequences) is an “erroneous treatment” zone. According to the POCT device regulation set out by the U.S. Food and Drug Administration (FDA), 95% of all measured blood glucose values must be within 15% of the true values, and 99% of measured values must be within 20% of the true values [28]. These results evidence the feasibility and accuracy of glucose detection using CA@PEG-2000 membranes, showing their potential for clinical diagnosis.

## 4. Conclusions

The present study developed a novel colorimetric analytical testing strip for whole blood glucose monitoring without sample pretreatment procedures. Three different types of cellulose acetate membranes were prepared using the phase separation technology, and the relationships between blood separation efficiency and porous structure were theoretically and experimentally investigated. CA@PEG-2000 membranes had an asymmetric structure with nano-pores on the surface and micro-pores in the interior, allowing for blood cell separation and plasma free-diffusion. Compared to conventional paper strips, the CA@PEG-2000 membranes exhibited high blood separation efficiency, short response time (less than 2 min), ultralow sample volume (5 μL), and high sensitivity. The fabricated pretreatment-free membranes can be applied to detect a variety of metabolites in whole blood, expanding the range of POCT rapid quantitative assays.

## Data Availability

The data presented in this study are available upon request from the corresponding author.

## Supplementary Materials

The following supporting information can be downloaded at https://www.mdpi.com/article/10.3390/bios12090712/s1, Table S1: The influences of different temperatures, the concentrations of CA, and types of solvent on diffusion distances; Figure S1: Linear calibration plot of the chromogenic intensity vs. glucose concentration using Image J software; Table S2: Performance comparison between our testing strip and other passive devices for plasma separation [29,30,31,32,33,34].
