# Supplementary material for: Micro-Volume Blood Separation Membrane for In-Situ Biosensing
Source: Biosensors (Basel). 2022 Sep 2;12(9):712. doi: 10.3390/bios12090712 (PMC9496035; doi:10.3390/bios12090712)
Supplement: Supplementary file 1 [file biosensors-12-00712-s001.zip › biosensors-1807504-supplementary.pdf]

Supplementary Materials

Micro−Volume Blood Separation Membrane for In−Situ Biosensing

Qin Zhu [1,2], Huimin Wu [3], Zhen Ma [2], Yuqiao Liu [2], Junmin Li [2], Ling Zhu [2], Xinran Zhang [2], Chengcheng Wang [2], Dajing Chen [2*] and Danhua Zhu [1*]

[1] State Key Laboratory for Diagnosis and Treatment of Infectious Diseases, The First Affiliated Hospital, Zhejiang University School of Medicine, Hangzhou 311100, China
[2] School of Pharmacy, Hangzhou Normal University, Hangzhou 311121, China
[3] MOE Key Laboratory of Macromolecular Synthesis and Functionalization, Department of Polymer Science and Engineering, Zhejiang University, Hangzhou 310027, China
[*] Correspondence: djchen@hznu.edu.cn (D.C.); zhudanhua@zju.edu.cn (D.Z.)

Table S1 The influences of different temperatures, the concentrations of CA, and types of solvent to diffusion distances.

Samples	Temperature (℃)	Solvent	Diffusion Distances (mm)
6% CA+6% PEG−2000	25	water	0
7% CA	25	water	1.5
7%CA+6%PEG−2000	15	water	2
7%CA+6%PEG−2000	20	water	3.1
7%CA+6%PEG−2000	25	water	4.5
7%CA+6%PEG−2000	35	water	4
7%CA+6%PEG−2000	45	water	2.3
7%CA+6%PEG−2000	25	Methanol :water 2:8	2
7%CA+6%PEG−2000	25	Methanol :water 3:7	0
7%CA+6%PEG−2000	25	Methanol :water 4:6	0

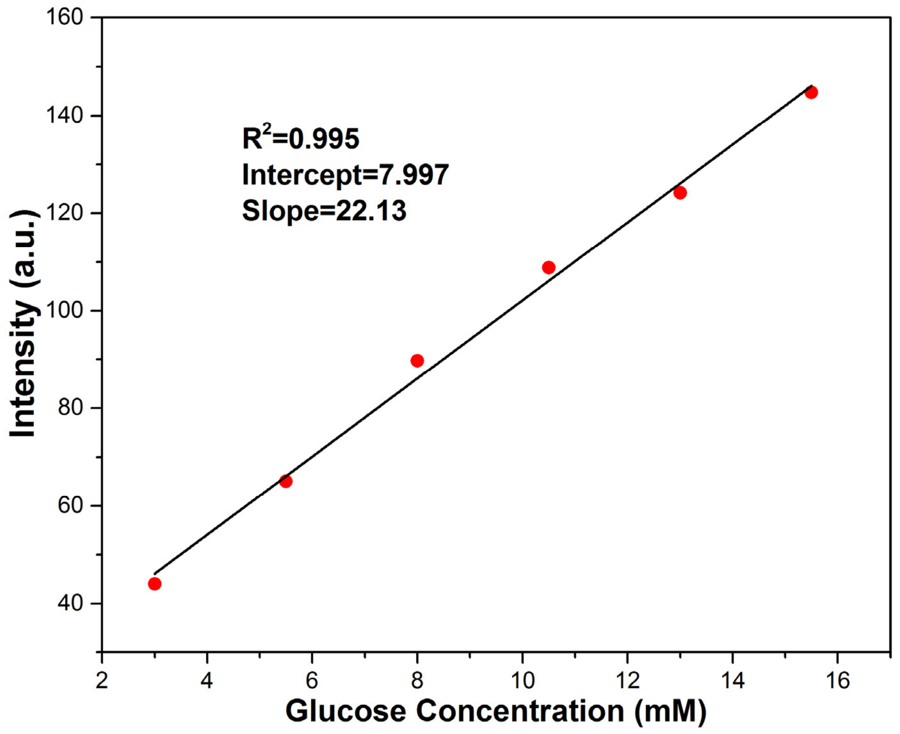

Figure S1. Linear calibration plot of the chromogenic intensity vs. glucose concentration using Image J software.

Table S2. Performance comparison between our testing strip and other passive devices for plasma separation.

References	Methods	Whole blood Volume	Separation Rate	Time(Process Rate)
[1]	Membrane filter	45 µl	60%	72 sec
[2]	Membrane filter	1.8 ml	28.6%–31%	
[3]	Membrane filter	250 µl	53.8%	10 min
[4]	Membrane filter	60 ml	71.7	6 min
[5]	microfluidic device	100 µl	20%–22%	10 min
[6]	microchip	200 µl	65%	25 min
This work	Membrane filter	5 µl	68.7%–75%	2 min

References

1. Xiao, Z.; Sun, L.; Yang, Y.; Feng, Z.; Dai, S.; Yang, H.; Zhang, X.; Sheu, C.; Guo, W.High-Performance Passive Plasma Separation on OSTE Pillar Forest. *Biosensors*. **2021**, *11*, 355
2. Liu, C.; Mauk, M.; Gross, R.; Bushman, F. D.; Edelstein, P. H.; Collman, R. G.; Bau, H. H. Membrane-based, sedimentation-assisted plasma separator for point-of-care applications. *Anal*

Chem. **2013**, *85*, 10463-10470.

3. Baillargeon, K. R.; Murray, L. P.; Deraney, R. N.; Mace, C. R. High-Yielding Separation and Collection of Plasma from Whole Blood Using Passive Filtration. *Anal Chem.* **2020**, *92*, 16245-16252.
4. Gao, Q.; Chang, Y.; Deng, Q.; You, H. A simple and rapid method for blood plasma separation driven by capillary force with an application in protein detection. *Anal Methods.* **2020**, *12*, 2560-2570.
5. Homsy, A.; van der Wal, P. D.; Doll, W.; Schaller, R.; Korsatko, S.; Ratzer, M.; Ellmerer, M.; Pieber, T. R.; Nicol, A.; de Rooij, N. F. Development and validation of a low cost blood filtration element separating plasma from undiluted whole blood. *Biomicrofluidics*. **2012**, **6**, 12804-128049.
6. Aota, A.; Takahashi, S.; Mawatari, K.; Tanaka, Y.; Sugii, Y.; Kitamori, T. Microchip-based Plasma Separation from Whole Blood via Axial Migration of Blood Cells. *Analytical Sciences*. **2011**, 27 (12), 1173-1178.
